# Good Sleep Quality Improves the Relationship Between Pain and Depression Among Individuals With Chronic Pain

**DOI:** 10.3389/fpsyg.2021.668930

**Published:** 2021-05-07

**Authors:** Zoe Zambelli, Elizabeth J. Halstead, Antonio R. Fidalgo, Dagmara Dimitriou

**Affiliations:** ^1^Sleep Education and Research Laboratory, Psychology and Human Development, University College London-Institute of Education, London, United Kingdom; ^2^Department of Psychology, University of East London, London, United Kingdom

**Keywords:** chronic pain, sleep quality, depression, moderation, pain interference, pain severity

## Abstract

Individuals with chronic pain often experience co-existing sleep problems and depression-related states. Chronic pain, sleep problems, and depression interrelate, and have been shown to exacerbate one another, which negatively impacts quality of life. This study explored the relationships between pain severity, pain interference, sleep quality, and depression among individuals with chronic pain. Secondly, we tested whether sleep quality may moderate the relationship between pain and depression. A cross-sectional survey was completed by 1,059 adults with non-malignant chronic pain conditions (*M*^*age*^ 43 years, 88% identified as women) and collected measures related to pain severity, pain interference, sleep quality, and depression. Multiple regression analyses found that pain severity, pain interference, and sleep quality are all significantly associated with depression. Secondly, moderated regression analyses revealed that sleep quality moderates the relationship between pain interference and depression among individuals with chronic pain such that good sleep quality attenuates the effect of pain interference on depression, and poor sleep quality amplifies the effect of pain interference on depression. These findings suggest that sleep quality may be a relevant therapeutic target for individuals with chronic pain and co-existing depression.

## Introduction

Chronic pain conditions are prevalent and burdensome across the globe. Depression is commonly reported among individuals with non-malignant chronic pain conditions which leads to impaired functioning and poor quality of life (IsHak et al., 2018). Estimates for depression among people with chronic pain have been reported between 20% and 60%, and evidence shows these individuals report a higher prevalence of depressive symptoms compared to a general population (Fishbain et al., 1997; Breivik et al., 2006; Rayner et al., 2016). Research has implicated several predictors of depression among this population including pain severity, pain interference, and illness perception (Costa et al., 2016). Furthermore, the relationship between chronic pain and depression may be bidirectional and lead to poorer health-related quality of life, and in some cases, longer duration of physical and psychological symptoms (IsHak et al., 2018). Evidence has also suggested that individuals with chronic pain and comorbid depression experience higher rates of physical, mental, and social dysfunction, and poorer response to pain treatments than chronic pain individuals without co-existing depression (Elliott et al., 2003; Holmes et al., 2013; Rayner et al., 2016). The co-existence of depression among chronic pain individuals further impacts the economy in addition to an individual's quality of life. Previous research conducted within NHS services found that among individuals with chronic pain, those who also met criteria for depression were more likely to be unable to work due to ill health, report higher levels of absenteeism, and that average health care costs for this group were higher compared to those without depression (Rayner et al., 2016). Notably, individuals from lower income backgrounds have been found to have lower participation rates in healthcare screenings which further compounds this issue. This may be due to having more difficulty understanding and applying health information, which over time contributes to higher healthcare costs (Chan et al., 2018; Svendsen et al., 2020).

Sleep problems are a significant complaint among individuals with chronic pain, with estimates of prevalence rates as high at 88% (Smith et al., 2001). These issues have been observed across different pain groups including musculoskeletal conditions, fibromyalgia, chronic headaches, and neuropathic pain conditions (Mathias et al., 2018). The most common sleep issues are insomnia (characterized by difficulty initiating sleep, frequent night awakenings, and early morning awakening), restless leg syndrome, and sleep disordered breathing (Call-Schmidt and Richardson, 2003; Mathias et al., 2018). Furthermore, the literature examining the relationship between pain and sleep problems points to a bidirectional relationship, with evidence to suggest that pain is predictive of sleep disturbance, and equally, poor sleep quality exacerbates pain outcomes, particularly among chronic pain individuals (Smith and Haythornthwaite, 2004; Andersen et al., 2018). In addition to pain, sleep disturbance is related to mood disorders. In particular, disturbed sleep is cited as a core symptom of depression, presenting in over 90% of clinically diagnosed individuals, and is included as a diagnostic criterion for Major Depressive Episode (MDE) in the Diagnostic and Statistical Manual for Mental Disorders (DSM-5) (Murphy and Peterson, 2015; Tolentino and Schmidt, 2018). Despite this, longitudinal research has established poor sleep as an independent risk factor for depression, indicating that the relationship between sleep and depression is likely reciprocal (Buysse et al., 2008; Jaussent et al., 2011; McCrae et al., 2016). Experimental studies have suggested that sleep may impact depression-related states through various mechanisms, including increased negative reactivity to stressful stimuli, amplifying negative mood, in addition to impairing frustration tolerance and suppressing emotional intelligence (Tempesta et al., 2010; Killgore, 2013; Finan et al., 2015; Killgore et al., 2017). In all, the evidence points to complex and intertwined relationships between pain, sleep, and depression-related states, and a limited number of studies have attempted to elucidate these further through theoretical and experimental studies.

Firstly, emerging science has implicated the role of neurobiological mechanisms which may explain the co-existence of chronic pain, poor sleep, and depression-related state. Notably, the role of proinflammatory cytokines have been shown to modulate spontaneous nociceptor activity and increase stimulus sensitivity which may lead to persistent pain states. Increased cytokine levels could also promote further secretion of new cytokines which consequently influence serotonergic neurotransmission implicated in the pathophysiology of depression. Finally, there is evidence that specific proinflammatory cytokines, namely interleukin-1 (IL-1) and tumor necrosis factor (TNF-α) act as sleep regulatory substances (Boakye et al., 2016). A recent study conducted by Rosseland et al. (2018) found that inducing sleep fragmentation led to increased pain sensitivity in a cohort of healthy adults, compared to one night of undisturbed sleep. In this study, researchers also tested whether an induced mood state (akin to depression-related states) would impact pain sensitivity and whether induced sleep and mood would interact to influence pain. Results showed neither induced negative mood on its own nor the interaction between induced sleep fragmentation and induced negative mood had any significant effect on pain. Furthermore, a 2-year longitudinal study demonstrated that prior pain was predictive of subsequent sleep disturbance among individuals with rheumatoid arthritis, and that those individuals with high pain levels and high sleep disturbance were at greater risk of developing symptoms of depression over time (Nicassio and Wallston, 1992). In another example, depression-related states were shown to mediate the relationship between poor sleep and pain interference (Ravyts et al., 2019).

Studies such as these have contributed toward developing evidence-based interventions and screening measures for chronic pain populations, however, there is still a need to examine these relationships further given the high prevalence of both sleep disturbance and depression in this group. Furthermore, it is important to note the role socio-demographic factors play on accessing screening and treatments to improve physical and psychological health outcomes. Socio-economic status (SES) is a crucial determinant of health and there is a need to measure SES more frequently in health and care research, particularly where chronic pain populations are concerned as many strategies to manage pain conditions rely on self-management approaches which are often negatively impacted by low SES (Marmot, 2017; Hardman et al., 2020).

Given the extent to which pain, sleep, and depression-related states co-exist, particularly among chronic pain populations, and the detrimental impact these have on individuals and society, elucidating the relationship between these factors could contribute toward improving outcomes for chronic pain individuals. In particular, this study sought to examine whether sleep quality acts as a moderator in the relationship between pain interference and severity, and depression, among a cohort of chronic pain individuals.

### Study Aims and Hypotheses

There were two aims of this cross-sectional study:

1. To establish whether pain interference, pain severity, and sleep quality are associated with depression, replicating previous research in this area
2. To examine whether sleep quality acts as a moderator in the relationship between pain (interference and severity) and depression, among individuals with chronic pain.

We hypothesized that pain interference, pain severity, and sleep quality would be associated with depression. Secondly, we hypothesized that sleep quality would indeed moderate the relationship between pain (inference and severity) and depression, specifically that high levels of sleep quality would attenuate the impact of high pain levels on depression.

## Methods

### Design

A cross-sectional survey was conducted between February and March 2020, before a government mandated lockdown to control the coronavirus pandemic. The survey was primarily hosted on Qualtrics (www.qualtrics.com), a survey management website, and paper copies were also available upon request to any potential participants unable to complete the survey via online means. This study was granted ethical approval by University College London, Institute of Education Ethics Committee. Participants were recruited through a social media campaign, and in collaboration with UK pain charities. Participants provided informed consent prior to completing the survey and did not receive compensation for taking part. Inclusion criteria for participants were adults aged 18 years and above, with a reported diagnosis of non-malignant chronic pain, and able to consent to participation of a survey study. Exclusion criteria were diagnoses of cancer-related chronic pain, and pain duration of <3 months.

### Participants

A total of 1,234 adult participants with self-reported chronic pain fully or partially completed an online or paper survey relating to their pain management, sleep, and mental health. After cleansing the data for complete responses, 1,059 fully completed surveys were included in this study. The majority of participants identified as women (88%), had a mean age of 42.88 years (*SD* = 13.25 years), and were of white ethnic origin (94%). In addition, nearly all (98%) reported a diagnosis of chronic pain via a healthcare professional (HCP), compared to 2% who self-diagnosed. Participants were asked to report their primary chronic pain condition, which were defined using the International Classification of Diseases (ICD) 11^th^ edition classification. Participants reported; Chronic Widespread Pain (CWP; 33%), Musculoskeletal (MSK; 35%), Headache and Orofacial (13%), Neuropathic (16%), and Visceral pain conditions (3%). Most participants reported using pain medication to manage their pain levels (91%) and over half reported their pain duration to be more than 10 years (57%). Finally, duration of sleep problems was more than 1 year for the majority of the group (87%). Participant characteristics are reported in Table 1.

**Table 1:** Participant characteristics.

**Variable**	***n***	**%**
Age^**^	42.88	13.25
**Gender**		
Men	126	12
Women	933	88
**Ethnicity**		
White-any background	933	94
Black/Black British	6	1
Asian/Asian British	12	1
Mixed	25	2
Other	23	2
**SEP**		
Very low	59	6
Low	275	26
High	323	30
Very high	402	38
**Primary pain condition**		
Chronic widespread pain	354	33
Musculoskeletal	373	35
Headache and orofacial	133	13
Neuropathic	164	16
Visceral	35	3
**Pain duration**		
<1 year	29	3
1–3 years	104	10
3–5 years	95	9
5–10 years	221	21
>10 years	602	57
Unsure	8	0
**Diagnosis**		
HCP diagnosed	1,037	98
Self-diagnosed	22	2
**Pain medication**		
Yes	959	91
No	100	9
**Sleep problem duration**		
<1 year	127	13
≥1 year	839	87

** *Age variable displays mean and standard deviation for a continuous variable*.

### Measures

Participants were required to complete background questions in addition to three validated questionnaires to measure pain interference, pain severity, sleep quality, and depression.

### Demographic Information

Participants were asked to report their diagnosis (self or via HCP) of non-malignant chronic pain. Demographic and socio-economic indicators included, age, gender, ethnicity, education attainment, employment status, household income, primary chronic pain condition, and pain medication status (see section Participants).

### Pain

The Brief Pain Inventory (BPI) short form is a widely used self-report measure for clinical pain (Cleeland and Ryan, 1991). The BPI includes two subscales which rate severity of pain and the degree to which pain interferes with common dimensions of feeling and function in the past 24 h. These are referred as (1) pain severity and (2) pain interference. Both subscales range from 0 to 10 with higher scores indicating higher levels of pain severity and pain interference. These scales have good internal consistency with Cronbach's alpha of 0.85 and 0.88 for the severity and interference scales, respectively (Tan et al., 2004).

### Sleep Quality

The Pittsburgh Sleep Quality Index (PSQI) is a validated measure for sleep research and consists of 24 items (Buysse, 1989). The scale comprises seven components which measure (1) subjective sleep quality, (2) sleep latency, (3) sleep duration, (4) sleep efficiency, (5) sleep disturbance, (6) daytime dysfunction, and (7) sleep medication over the past month. Each component generates a score from 0 to 3 where higher scores indicate poorer sleep outcomes. A sum of the seven component scores can be used to generate a global PSQI score ranging from 0 to 21. A global score above five indicates poor sleep quality. The PSQI has been validated among individuals with chronic pain conditions with Cronbach's alpha scores above 0.7 (Nicassio et al., 2014).

### Depression

Depression was measured using the Hospital Anxiety and Depression Scale (HADS). A 14-item validated measure designed to measure anxiety and depression symptoms during the past week (Zigmond and Snaith, 1983). It comprises of two subscales assessing anxiety (HADS–A) and depression (HADS–D). Items are rated on a four-point Likert scale (e.g., 0 = not at all to 3= most of time). Five items require reverse scoring. Scores for each item are summed for each subscale, scores above eight suggest anxiety and depression with thresholds described as scores between 0 and 8 = no symptoms, 8 and 10 = mild symptoms, 11 and 14 = moderate symptoms, 15 and 21= severe symptoms (Zigmond and Snaith, 1994; Stern, 2014). The HADS-D has been validated among individuals with chronic pain conditions with Cronbach's alpha scores of 0.8 (Turk et al., 2015).

### Analyses

The final sample was reduced to 1,059 participants after removing participants with missing data for the pain, sleep, depression, and demographic variables. All analyses were conducted on this sample who had a fully completed data set. A variable was created to calculate socio-economic position (SEP) using education, employment, and household income (Halstead et al., 2018). Education attainment was split into 0 = secondary school education or below and 1 = higher ed, undergraduate, and postgraduate education, employment status was split 0 = not employed and 1 = employed full or part time, finally, household income was scored 0 = low income ≤£20,000 and 1 ≥£20,000 (Cribb et al., 2018). Total SEP was calculated by summing the scores of these three indicators from the dichotomous coding, where 0 = very low and 3 = very high SEP (see Table 1).

Normality tests were conducted on the four main variables (pain severity, pain interference, sleep quality, depression). Descriptive analyses examined the means, standard deviations, and minimum to maximum values across the main variables (pain interference, pain severity, sleep quality, and depression), and where possible, frequencies and percentages for clinical cut-offs across the sample. A simple regression looked at the association between the components of the sleep quality measure (PSQI) and depression (HADS-D). *T*-tests were conducted to examine whether there were any differences in pain, sleep, or depression scores based on gender, diagnosis type, and pain medication type. To avoid multicollinearity between pain interference and pain severity subscales, two linear regressions (model A: pain severity and model B: pain interference) examined the relationships of all predictor variables (pain severity, pain interference, sleep quality) and demographic indicators (age, gender, SEP, pain condition group) on depression. Significant associations between demographic variables and depression were then included in the following moderated regression models as covariates.

**Figure 1:**
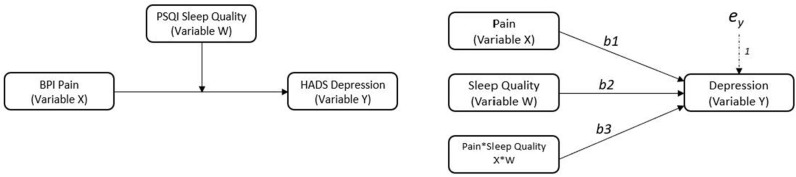
Conceptual and statistical models showing sleep quality as a moderator on the relationship between pain and depression. Variable X is the predictor variable, variable W is the moderator, variable Y is the outcome.

To examine sleep quality as a potential moderator, two moderated regression analyses were conducted. The “PROCESS” macro and customs dialogue box was installed into SPSS v. 26 to conduct moderated regression analyses (Hayes, 2017). In the first analysis (model 1), pain severity, sleep quality, and demographic covariates were entered as predictors along with an interaction term between pain severity and sleep quality. In the second analysis (model 2), pain interference, sleep quality, and demographic covariates were entered as predictor variables along with an interaction term between pain interference and sleep quality. Continuous predictor variables were mean centered in both models (i.e., the variable mean is subtracted from every value of the variable). Figure 1 illustrates the moderated regression models.

## Results

### Descriptive Statistics and Preliminary Analyses

The mean, standard deviation, minimum, and maximum scores for each measure are displayed in Table 2, as well as frequencies and percentages for clinical cut-offs, where available. A full range of scores were observed across all main variables, with the exception of sleep quality, where no participants scored between 0 and 2 which would indicate very good sleep quality. Further to this, only 3% of the sample scored within range for “good sleep quality,” with 97% of participants in range for “poor sleep quality” based on scores above five (Buysse, 1989). Less than half the sample scored within “normal” range for depression (indicating no symptoms), and 63% scored within range for either “mild,” “moderate,” or “severe” depression symptoms (Stern, 2014).

**Table 2:** Means, standard deviations, and minimum to maximum scores for all variables.

**Variable**	**Subscale**	**Mean**	***SD***	**Min–Max**
BPI pain				
	Pain interference	6.36	2.21	0–10
	Pain severity	5.46	1.86	0–10
PSQI sleep quality		13.89	3.79	2–21
HADS depression		9.19	4.44	0–21
PSQI cut-off		***N***	**%**	
	Good sleep quality	27	3	
	Poor sleep quality	1,032	97	
HADS-D cut-off				
	Normal	389	37	
	Mild	287	27	
	Moderate	246	23	
	Severe	137	13	

*BPI, Brief Pain Inventory; PSQI, Pittsburgh Sleep Quality Index; HADS, Hospital Anxiety and Depression scale; Cut-offs provided for PSQI and HADS measures only*.

Results of a simple regression to explore the associations between the seven components of the sleep quality measure and depression are displayed in Table 3: *R*^2^ = 0.22, *F*_(7, 1, 051)_ = 41.24, *p* ≤ 0.001. Subjective sleep quality β = 0.11*, p* = 0.01, daytime dysfunction β = 0.24*, p* ≤ 0.001, and global sleep quality β = 0.26, *p* = 0.05 were positively and significantly associated with depression.

**Table 3:** Associations between seven PSQI components and global sleep quality score on depression.

**PSQI component**	**Beta (β)**	***p***
***R*** **=** **0.46**, ***R***^**2**^ **=** **0.22**, ***F*** **=** **41.24**, ***p*** **≤** **0.001**		
Subjective sleep quality	0.11	**0.01**
Sleep latency	0	0.99
Sleep duration	−0.08	0.27
Sleep disturbance	0.05	0.24
Sleep medication	−0.5	0.33
Daytime dysfunction	0.24	** <0.001**
Global sleep quality	0.26	**0.05**

*Bold text displays results from the regression analysis*.

*T*-tests concluded there were no significant gender differences for pain or sleep outcomes, however it was found that men had slightly higher depression scores than women *t*_(1, 057)_ = 2.19, *p* = 0.03, *d* = 0.23. Furthermore, there were no differences in pain severity, pain interference, or depression scores among formally diagnosed participants (via HCP) compared to self-diagnosed, and sleep quality scores were marginally higher (signifying poorer sleep quality) among those with a formal diagnosis compared to the self-diagnosed group *t*_(1, 057)_ = 2.00, *p* = 0.49, *d* = 0.42. Finally, individuals taking pain medication in order to control pain symptoms, had statistically poorer sleep quality *t*_(1, 057)_ = −2.66, *p* = 0.008, *d* = 0.28 and marginally higher depression *t*_(1, 057)_ = −1.93, *p* = 0.054, *d* = 0.20 than individuals taking no pain medication.

When examining the linear regressions, model A revealed significant main effects of pain severity, sleep, SEP, and MSK pain on depression *R*^2^ = 0.20, *F*_(8, 1, 050)_ = 32.46, *p* = 0.001. Model B revealed significant main effects of pain interference, sleep, gender, SEP, and chronic widespread pain on depression *R*^2^ = 0.29, *F*_(8, 1, 050)_ = 54.23, *p* = 0.001. Non-significant variables were excluded from the subsequent moderated regression models.

### Moderation Regression Analyses

Moderated regression analyses were conducted to test whether sleep quality would moderate the relationships between pain severity and depression (model 1) and pain interference and depression (model 2), among individuals with chronic pain conditions. Results are displayed in Table 4.

**Table 4:** Moderated multiple regression for pain severity and pain interference as predictor variables and sleep quality as moderator variable on depression.

	***b***	***t***	***p***
***R*** **=** **0.44**, ***R***^**2**^ **=** **0.19**, ***F*** **=** **50.75**, ***p*** **≤** **0.001**			
BPI pain severity	0.31	4.14	<0.001
PSQI sleep quality	0.34	9.45	<0.001
Pain severity * sleep quality	0.01	0.72	0.474
SEP	−0.74	−5.30	<0.001
MSK pain	−0.65	−2.50	0.013
***R*** **=** **0.54**, ***R***^**2**^ **=** **0.29**, ***F*** **=** **72.76**, ***p*** **≤** **0.001**			
BPI pain interference	0.77	12.8	<0.001
PSQI sleep quality	0.22	6.24	<0.001
Pain interference * sleep quality	0.03	2.20	0.028
Gender	−0.77	−2.14	0.033
SEP	−0.50	−3.84	<0.001
Chronic widespread pain	0.58	2.34	0.020

*Bold text displays results from the moderated multiple regression analyses*.

Model 1 accounted for a significant amount of variance in depression; *R*^2^ = 0.19, *F*_(6, 1, 052)_ = 50.75, *p* ≤ 0.001. Pain severity, sleep quality, SEP, and MSK pain were all independently significantly associated with depression. However, the interaction between pain severity and sleep quality was non-significant.

Model 2 accounted for a significant amount of variance in depression; *R*^2^ = 0.29, *F*_(6, 1, 052)_ = 72.76, *p* ≤ 0.001. Pain interference, sleep quality, gender, SEP, and chronic widespread pain were all independently significantly associated with depression. In this model, there was a significant interaction between pain interference and sleep quality; Δ*R*^2^ = 0.003, Δ*F*_(1, 1, 052)_ = 2.82, *p* = 0.028. Following recommendations by Aiken et al. (1991) an interaction plot was created to aid interpretation of the interaction.

**Figure 2:**
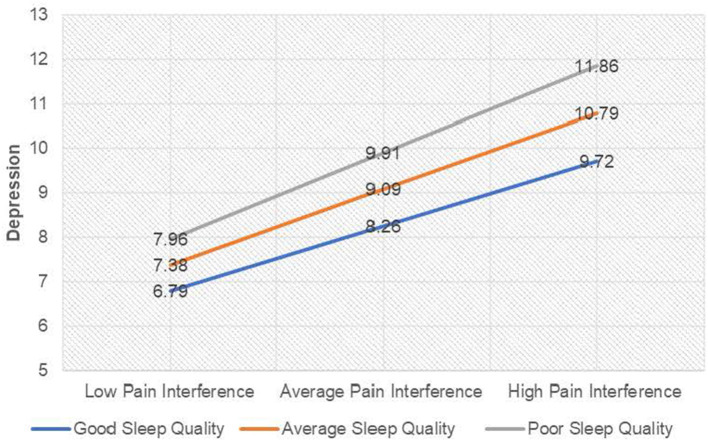
Interaction plot for depression among individuals with chronic pain. For ease of interpretation, sleep quality labels have been raised. “Good sleep quality” as labeled on the graph denotes a score ISD below the mean on the PSQI (lower scores on the PSQI indicate better sleep quality), and “Poor sleep quality” denotes a score ISD above the mean on the PSQI (higher scores on the PSQI indicate poor sleep quality).

Visual inspection of Figure 2 demonstrated there was a positive relationship between pain interference and depression (as pain interference scores increased, depression scores increased) when sleep quality scores were one standard deviation below the average; *b* = 0.66, 95%CI [0.52, 0.80], *t* = 9.04, *p* ≤ 0.001, when sleep quality scores were average; *b* = 0.77, 95%CI [0.65, 0.89], *t* = 12.80, *p* ≤ 0.001, and when sleep quality scores one standard deviation above the average; *b* = 0.88, 95%CI [0.72, 1.04], *t* = 10.60, *p* ≤ 0.001. Taken together, it can be suggested that good sleep quality attenuated the effect of pain interference on depression scores and equally poor sleep quality amplified the effect of pain on depression scores.

## Discussion

The purpose of this study was to investigate variables associated with depression among a cohort of chronic pain individuals and examine whether sleep quality can moderate the effect of pain severity on depression, and pain interference on depression, among a chronic pain population. Our findings suggest that pain severity, pain interference, and sleep quality are all significantly associated with depression among this population. These results support previous research which posits pain is an antecedent to depression, and several studies which have implicated sleep quality as an initiating factor in the development of depression (Magni et al., 1994; Chang et al., 1997; Fishbain et al., 1997; Buysse et al., 2008; Jaussent et al., 2011).

Our findings also indicate that sleep quality moderates the relationship between pain interference and depression, such that better sleep quality buffers the effect of pain interference on depression, and equally poorer sleep quality exacerbates the impact of pain interference on depression in this group. This evidence supports one study conducted by Hamilton et al. (2007) in which they found sleep quality moderated the effect of pain intensity on negative affect (akin to depression) among rheumatoid arthritis and fibromyalgia patients. In addition to supporting this evidence, our study amplifies these findings among a larger sample size (*n* = 1,059 vs. *n* = 49) and suggests the moderating role of sleep quality may be found across several non-malignant pain groups.

Examining the relationships between pain, sleep, and depression in chronic pain populations is an important area of research, and studies have previously focused on depression-related states (e.g., negative affect, negative mood, depression) as a moderating factor between pain and sleep (Valrie et al., 2008; O'Brien et al., 2011). Subsequently, some researchers have advocated for targeting depression-related states as a therapeutic intervention among this group as a means to improve sleep quality, and in some instances, pain symptoms. Despite this, two issues arise with this approach: firstly, antidepressant medications (predominantly used in moderate to severe cases of depression) have shown to improve sleep parameters such as sleep latency and increase slow wave sleep in some instances and are recommended to be combined with a behavioral intervention (Schmid et al., 2006; Wichniak et al., 2017). However, the sleep-promoting action provided by certain antidepressants may become problematic during the maintenance phase of treatment due to over-sedation effects. Additionally, some medications used to treat depression often result in sleep disruption, whether through activating agents or through long-term increased tolerance to sedatives (Wichniak et al., 2017). Secondly, in a study which compared a behavioral intervention for sleep to a behavioral intervention for depression among individuals with both insomnia and depression, results showed that both interventions produced reductions in depression symptoms post-intervention and 3-year follow-ups, although these reductions did not differ across the two intervention groups. In addition, the group receiving the sleep treatment demonstrated significantly better improvements in sleep parameters than the depression intervention (Blom et al., 2017). The authors concluded therefore that in individuals with co-existing depression and insomnia, a behavioral sleep intervention should be included as a treatment option in addition to any pharmaco-therapy or psychological treatment for depression. Given that sleep problems are shown to be a precipitating and maintaining factor in depression, targeting depression through pharmacologic and non-pharmacologic therapies solely whilst neglecting co-existing sleep problems may only lead to short-term benefits (Tsuno et al., 2005). Several studies suggest talking therapies such as cognitive behavioral therapy for insomnia (CBT-I) are an effective intervention in reducing not only insomnia, but also improving depression outcomes significantly among clinical populations (Manber et al., 2011, 2016; Gee et al., 2019). The UK National Institute for Health and Care Excellence (National Institute for Health and Clinical Excellence, 2009) provide guidelines for treatment of depression which advocate a stepwise approach based on severity and duration of symptoms. Within these guidelines, sleep hygiene is advised for individuals with subthreshold depressive symptoms and those with recognized mild to moderate cases, and our study supports the notion that in addition to sleep hygiene, a more structured approach such as CBT-I may be considered.

Nearly all our sample met criteria for poor sleep quality (determined by the PSQI measure) and average sleep quality scores were significantly above the cut-off for “poor” sleep quality (mean PSQI scores were 13.89, nearly nine points above the cut-off of five). Our findings are similar to previous studies using this measure among chronic pain populations (Smith et al., 2000; Osorio et al., 2006; Marty et al., 2008). In addition, a significant portion of our sample also met criteria for mild, moderate, and severe depression symptoms. To contextualize, our findings suggested there is an association between sleep quality and depression and supports the notion that focusing on sleep problems could help alleviate both symptoms of insomnia and depression, as others have done previously (Asarnow and Manber, 2019). Given that depression has been shown to impact self-management, opioid use, and health-seeking behavior among chronic pain populations, it is important to both screen for and therapeutically address sleep problems which may influence depression in this group (Jordan et al., 2006; Bair et al., 2009; Goesling et al., 2015).

Lastly, we included socio-demographic covariates in our models which were found to be significantly associated with depression among our cohort. Socio-economic position (SEP) was significant in both moderated regression models and suggests that chronic pain individuals from lower SEP backgrounds are at greater risk of experiencing comorbid depression. This supports previous findings and strengthens the basis for not only recording SEP during interactions with health and social care systems, but screening for SEP and tailoring health messaging to these groups (Freeman et al., 2016; Booher, 2019). Given the correlation between low SEP and low health-literacy rates, one strategy proposed has been to simplify text within health messaging and include illustrations where possible (Houts et al., 2006; Wilson and Wolf, 2009; Svendsen et al., 2020).

### Limitations

Our study relied on self-report measures which may be less sensitive than objective measures and comprehensive clinical assessments. Despite this, self-report measures offer an effective means to data gathering across larger samples (Paulhus and Vazire, 2007). Secondly, the use of cross-sectional data limits the ability to establish temporal relationships and make causal inferences regarding pain sleep and depression. However, principal works in this field have shown that these relationships are likely reciprocal, and consequential of one another. Therefore, our results may still be meaningful in enhancing care pathways for chronic pain populations. Third, our population consisted of a community dwelling sample, which likely includes individuals with a wide range of health needs. Future research should seek to replicate this model among patients within clinical settings to assess whether the findings are replicated among those with the most complex needs. Fourth, the majority of our sample included participants who identified as women from a “White-any” ethnic background which limits generalisability of our findings to other demographic groups with chronic pain. Finally, it is possible that the association between sleep and depression may be influenced by the use of sleep-influencing medications among chronic pain populations and future research could examine this as a potential control variable.

### Recommendations for Research and Practice

Given the high prevalence and impact depression has on quality of life, self-management care, and economic productivity among individuals with chronic pain, seeking strategies to reduce comorbid depression in this population is of great value. Our findings suggest sleep quality is associated with depression in this group. In light of our findings, and supported by previous research, the following recommendations are made:

1. Given that sleep quality may moderate the impact of pain interference on depression, chronic pain services should regularly include screening for sleep problems and symptoms of depression during routine assessments.
2. NICE guidelines (National Institute for Health and Clinical Excellence, 2009) for depression include sleep hygiene advice for individuals with persistent subthreshold depression, mild and moderate cases. More structured sleep interventions such as CBTI could be considered within combined treatment approaches for individuals with co-occurring sleep problems and depression.
3. Practitioners and social care workers should be encouraged and supported to screen for SEP and address specific barriers to treatment uptake among lower SEP individuals to further improve depression outcomes in this population e.g., telehealth solutions which minimize need and expense for travel.

## Data Availability Statement

The raw data supporting the conclusions of this article will be made available by the authors, without undue reservation.

## Ethics Statement

The studies involving human participants were reviewed and approved by UCL Institute of Education Ethics Committee. The patients/participants provided their written informed consent to participate in this study.

## Author Contributions

ZZ and EH: concept and study design and data analyses, ZZ: survey creations and data cleansing. ZZ, EH, AF, and DD: manuscript and editing. All authors contributed to the article and approved the submitted version.

## Conflict of Interest

The authors declare that the research was conducted in the absence of any commercial or financial relationships that could be construed as a potential conflict of interest.
